# The Parent Psychological Flexibility Questionnaire (PPFQ): Item Reduction and Validation in a Clinical Sample of Swedish Parents of Children with Chronic Pain

**DOI:** 10.3390/children3040032

**Published:** 2016-11-19

**Authors:** Camilla Wiwe Lipsker, Marie Kanstrup, Linda Holmström, Mike Kemani, Rikard K. Wicksell

**Affiliations:** 1 Functional Area Medical Psychology/Functional Unit Behavior Medicine, Uppgång P8:01, Karolinska University Hospital, SE-171 76 Stockholm, Sweden; marie.kanstrup@ki.se (M.K.); linda.holmstrom@ki.se (L.H.); mike.kemani@ki.se (M.K.); rikard.wicksell@ki.se (R.K.W.); 2 Department of Clinical Neuroscience, Karolinska Institutet, 171 77 Stockholm, Sweden; 3 Department of Women’s and Children’s Health, Karolinska Institutet, 171 77 Stockholm, Sweden

**Keywords:** psychological flexibility, validation, pediatric chronic pain, parents, anxiety, depression, distress, Acceptance and Commitment Therapy (ACT)

## Abstract

In pediatric chronic pain, research indicates a positive relation between parental psychological flexibility (i.e., the parent’s willingness to experience distress related to the child’s pain in the service of valued behavior) and level of functioning in the child. This points to the utility of targeting parental psychological flexibility in pediatric chronic pain. The Parent Psychological Flexibility Questionnaire (PPFQ) is currently the only instrument developed for this purpose, and two previous studies have indicated its reliability and validity. The current study sought to validate the Swedish version of the 17-item PPFQ (PPFQ-17) in a sample of parents (*n* = 263) of children with chronic pain. Factor structure and internal reliability were evaluated by means of principal component analysis (PCA) and Cronbach’s alpha. Concurrent criterion validity was examined by hierarchical multiple regression analyses with parental anxiety and depression as outcomes. The PCA supported a three-factor solution with 10 items explaining 69.5% of the total variance. Cronbach’s alpha (0.86) indicated good internal consistency. The 10-item PPFQ (PPFQ-10) further explained a significant amount of variance in anxiety (29%), and depression (35.6%), confirming concurrent validity. In conclusion, results support the reliability and validity of the PPFQ-10, and suggest its usefulness in assessing psychological flexibility in parents of children with chronic pain.

## 1. Introduction

Psychological flexibility, the core treatment target in Acceptance and Commitment Therapy (ACT) [1], is conceptualized as the ability for adaptive, values-oriented behavior in the presence of distressing experiences [2]. Psychological flexibility has also been described as a “fundamental aspect of health”, with high relevance to distress, such as anxiety and depression, where there is typically a lack of flexibility [3]. Current research further indicates that parental psychological flexibility may act as a possible moderator in the relationship between parent- and child-distress [4], and also that psychological flexibility within the parenting role is associated with adaptive parenting behavior in non-clinical samples [4,5].

There is emerging evidence for the utility of ACT in pediatric chronic pain [6,7,8,9]. In the context of pediatric chronic pain, parental psychological flexibility is defined as the parent’s willingness to experience distress related to the child’s pain, in the service of long-term values and related behavioral goals for both parent and child [10,11]. Research on parents of children with chronic pain has also shown a positive relationship between parental psychological flexibility and level of functioning in the child, and has pointed to the utility of targeting parental psychological flexibility in treatments with ACT for pediatric chronic pain, in order to potentially improve child treatment effects [10,11]. There is also mounting evidence concerning the association between parental distress, such as anxiety, fear, catastrophizing, and depression, and functional disability in children with chronic pain [12,13,14], even at subclinical levels of parental distress [15]. For example, research shows that parental distress influences parent behavior toward more protecting and monitoring, which contributes to the child’s distress, fear, catastrophizing, and avoidance behaviors; factors with a direct impact on pain related functional disability in the child [16,17,18].

Psychological flexibility appears to be a context specific construct, in terms of the area of study (e.g., pain, parenting, workplace, or other), and therefore needs to be assessed as such [4,19]. This implies the need for reliable and valid instruments to assess parental psychological flexibility within the context of pediatric chronic pain. To date, only one such instrument has been specifically developed, the Parent Psychological Flexibility Questionnaire (PPFQ) [10,11]. However, the PPFQ has not yet been validated in a Swedish sample. The first study by McCracken and Gauntlett-Gilbert included development of the PPFQ by generating a set of items constructed to reflect aspects related to psychological flexibility (e.g., acceptance, cognitive defusion, and values-based action), of which 24 of the original 31 items were retained for analysis. Results supported internal reliability for the preliminary instrument and suggested concurrent validity through associations with child functioning and parent responses to child pain [11]. The second study by Wallace et al. [10] aimed to further refine and examine the reliability and validity of the 24-item PPFQ (PPFQ-24). This resulted in a 17-item questionnaire (PPFQ-17) with four factors: (1) values-based action (VBA); (2) emotional acceptance (EA); (3) pain acceptance (PA); and (4) pain willingness (PW). The subscales and the full measure generally had adequate internal reliability (though weaker for the three-item PW subscale), and demonstrated adequate correlations with adolescent-reported measures of pain, functioning, pain acceptance, and distress. The authors concluded the PPFQ-17 to be a reasonably reliable and valid measure for clinical use and further research. However, it was also noted that the PA subscale, with few significant correlations and “no unique contributions within the measures included”, should be further investigated in future studies designed to test, for example, parental distress, which may result in a briefer measure excluding the PA subscale [10]. Therefore, the objectives of the current study were to (1) explore the factor structure and reliability of the Swedish version of the PPFQ-17 in a sample of parents of children referred to a tertiary pain clinic for chronic pain; and (2) to investigate the concurrent validity of the PPFQ by analyzing its relation to parental distress (anxiety and depression) among parents of children and adolescents with chronic debilitating pain.

## 2. Materials and Methods

### 2.1. Procedure and Participants

The study utilized a cross-sectional design including 263 parents of children with chronic pain who had been consecutively referred to a tertiary care pain clinic in Stockholm County, Sweden. Participants were recruited during a period of four years, and were excluded from the study if they were non-Swedish speakers. Mean age for the participating parents was 44.4 years, and the sample consisted of 81% women. A majority of the participating parents (53%) reported having studied at the university level. The regional ethical review board in Stockholm (Regionala etikprovningsnamnden i Stockholm; FE 289; 171 77; Stockholm, Sweden) approved of the study and participants provided written and informed consent. Assessments of the children were also collected, and analyses and results based on these assessments have been previously reported [20,21]. In brief, most children reported multisite pain (76%), the mean pain duration was four years, the majority of the children were girls (72%), and the mean age was 14 years (*SD* = 2.64).

### 2.2. Assessments

Data was collected by means of self-report questionnaires by the parent accompanying the child to the first visit at the clinic prior to any assessment or intervention.

#### 2.2.1. Demographic Variables

Demographic variables included parental age, parental gender, and parental level of education divided into two categories: basic education/high school and university studies.

#### 2.2.2. Distress (Anxiety and Depression)

The Hospital Anxiety and Depression Scale (HADS) was used to assess anxiety and depression in parents. The two subscales, anxiety (HADS-a) and depression (HADS-d), each consist of seven items rated on a four-point Likert scale (0–3) [22]. The scale has established clinical cutoffs at a score of 8 for each scale [23], and the HADS is one of the most widely used measures of anxiety and depressive symptoms in general medical settings and has been used with a wide variety of medical patients [24]. It has also been used in a number of validation, intervention, and correlational studies concerning acceptance and psychological flexibility, in relation to chronic pain, but also for other conditions as well [25,26,27,28,29,30,31]. Furthermore, it has been used with parents of adolescents with chronic illness and, compared with other measures of anxiety or depression, it excludes symptoms that might be somatic aspects of physical illness, such as, for example, insomnia or fatigue [32]. In the current sample, Cronbach’s alpha was 0.88 for both subscales (HADS-a and HADS-d).

#### 2.2.3. Parental Psychological Flexibility

The PPFQ aims to assess parents’ ability to effectively manage their own distress concerning their adolescent’s pain, while keeping actions directed towards goals and values [10,11]. The version used in the current study contained the 17 items as proposed by Wallace et al. [10] and four subscales (factors) as described above, and parents responded to each item using a seven-point scale ranging from 0 = never true to 6 = always true. Items were developed to reflect aspects of relevance to psychological flexibility [2], namely acceptance, cognitive defusion, and values-based action. Cronbach’s alpha for the original 24-item scale was 0.91, and this scale was validated by comparison with demographic variables, pain related variables, adolescent functioning, and parent responses to child pain. Initial analyses suggested the reliability and validity of the scale regarding parent behavior, as well as its potential importance in relation to adolescent functioning [11]. Cronbach’s alpha for PPFQ-17 was 0.87 in the original study by Wallace et al. [10] and 0.90 in the current sample.

### 2.3. Translation and Swedish Adaptation

Permission to translate and validate the PPFQ from the English version, originally developed by McCracken et al. [11], to Swedish, was obtained from the authors of the original PPFQ. Based on standards for self-report translation and cross-cultural adaption processes [33], the PPFQ was translated by a clinical psychologist specialized in chronic pain and ACT, with a PhD in clinical psychology, and back-translated by two proficient English speakers. Prior to the study, the pre-final version was administered to a group of parents of children with chronic pain. They provided input on the wording and content of the questionnaire and were asked about possible difficulties related to understanding item-content.

### 2.4. Analytical Approach

#### 2.4.1. Data and Missing Values Analysis

All analyses were computed using SPSS Statistics (Version 22; IBM: Armonk, NY, USA, 2013). Frequency distributions were analyzed to ascertain multivariate normality of data. Analysis of missing values was performed, and the Expectation-Maximization algorithm (E-M) for imputing missing data was employed.

#### 2.4.2. Factor Structure and Internal Consistency

Initial analyses included inter-item correlations, sampling adequacy, and factorability of the PPFQ-17 [34]. In line with the aim of further exploration, rather than testing the model fit of the existing factor solution, a principal components analysis (PCA) was performed to examine the underlying factor structure of PPFQ items, and to optimize the possibility for data reduction [34]. Both varimax (orthogonal) and direct oblimin (oblique; with delta = 0) rotations of the factor loading matrix were used to achieve the simplest factor structure possible [35], before deciding on a rotation for the final model. The rotated factor structure was examined for items with poorer extraction (communality; lower than 0.4) within the factor structure or cross-loadings (higher than 0.32 on a second factor) to determine the most simple solution with clear and meaningful factors [35]. The internal consistency of the total scale and the respective subscales was calculated using Cronbach’s alpha in an iterative process in order to identify items that did not contribute, or had a negative contribution, to the scales’ alpha. Items with a negative impact were subsequently removed. The strength of Cronbach’s alpha was determined according to the reliability matrix for research in psychology by Ponterotto and Ruchdeschel [36].

#### 2.4.3. Concurrent Criterion Validity

The concurrent criterion validity of the PPFQ (subscales and total scale) was investigated by analyzing the relationship with anxiety (HADS-a) and depression (HADS-d). Pearson product-moment correlation coefficients were calculated between demographic variables, psychological flexibility, anxiety, and depression. A correlation *r* = ±0.00–0.29 was considered weak, *r* = ±0.30–0.49, moderate, *r* = ±0.50–0.89, strong, and *r* ≥ ±0.90, very strong [37]. Following correlations, two hierarchical regression analyses were performed to investigate the variance explained by PPFQ in anxiety and depression respectively. Age, gender, and education were included as control variables in the first step (step 1), followed by the PPFQ (step 2).

## 3. Results

### 3.1. Data and Missing Values Analysis

Of the 280 participants recruited, 94% (*n* = 263) had data on the main variables included (i.e., PPFQ and HADS) and could therefore be used for the analyses. Across all parent forms, 30 data points were missing for the PPFQ (0.8% of possible responses), and 18 data points were missing for HADS (0.5% of possible responses). Missing data was found to be missing completely at random (MCAR) [38]. Visual inspection of histograms, Q-Q plots, and box plots showed that the scores on the main variables were approximately normally distributed.

### 3.2. Exploratory Factor Analysis

All 17 PPFQ-items correlated at least 0.3 with at least one other item, and no item correlated with another item above 0.8, suggesting reasonable factorability. Communalities were all above 0.3, indicating that items shared some common variance with other items. The Kaiser-Meyer-Olkin measure of sampling adequacy was 0.89, and Bartlett’s test of sphericity was significant (χ^2^ (136) = 2263.964, *p* < 0.000). The diagonals of the anti-image correlation matrix were all over 0.5.

All 17 PPFQ-items were subjected to a PCA. Three components achieved eigenvalues greater than 1 (the initial five eigenvalues were 6.68, 2.23, 1.38, 0.94, and 0.84), and inspection of the scree plot also suggested a three-factor solution. Based on previous research resulting in four factors, three- and four-factor solutions were examined using both varimax and oblimin rotations of the factor loading matrix. The three-factor solution, which explained 60.5% of the variance in the PPFQ-17, was finally chosen based on eigenvalues and the pattern in the scree plot, and because there were an insufficient number of primary loadings and difficulties in interpreting the fourth factor. We subsequently repeated the analysis with a forced extraction of three factors. Since there was little difference between the varimax and oblimin solutions, we decided on an oblimin rotation for the final solution, assuming that the latent factors would be related [34].

In order to determine as simple a solution as possible, with clear and meaningful factors and best possible internal consistency, items were then sequentially eliminated due to loading on multiple factors, poorer extraction (communality) within the factor structure, or negative contribution to each subscale’s alpha, according to the criteria detailed in Section 2.4.2. This process resulted in a 10-item measure (PPFQ-10) with three factors that explained 69.5% of the variance (Table 1). All factor loadings were greater than 0.68, and each subscale had a number of items loading strongly upon it. Three of the four-factor labels proposed by Wallace et al. [10], VBA, PW, and EA, suited the three extracted factors perfectly and were subsequently retained. In line with the previous discussion in Wallace et al. [10], one of the factors, PA, was eliminated in the process. Internal consistency for each of the scales was examined using Cronbach’s alpha, taking into account the number of items [36]. The subscales had fair, moderate, and excellent internal consistency (PW, VBA, EA, respectively, α between 0.73 and 0.87), and the full measure had good internal consistency (α = 0.86). The skewness and kurtosis were within a tolerable range for assuming a normal distribution, and examination of the histograms further suggested that the distributions were approximately normal. Alpha values and descriptive statistics are presented in Table 2.

### 3.3. Concurrent Criterion Validity

#### 3.3.1. Descriptive Analyses and Correlations

Means, standard deviations, and bivariate correlations for all variables are presented in Table 3. Regarding parental distress, as measured by the HADS (with clinical cutoffs of 8 for each scale) [23], 35% of parents reported levels of anxiety symptoms at 8 or above and 21% reported depressive symptoms at 8 or above.

There was a significant positive relationship between the PPFQ-17 and the PPFQ-10 versions (*r* = 0.96, *n* = 263, *p* < 0.000). This correlation was very strong. All three PPFQ-10 subscales had a significant positive and strong correlation with both the PPFQ-10 and PPFQ-17 full scales. Furthermore, results revealed significant negative correlations between psychological flexibility and anxiety (PPFQ-17: *r* = −0.48, *n* = 263, *p* < 0.000; PPFQ-10: *r* = −0.52, *n* = 263, *p* < 0.000), and between psychological flexibility and depression (PPFQ-17: *r* = −0.49, *n* = 263, *p* < 0.000; PPFQ-10: *r* = −0.53, *n* = 263, *p* < 0.000). Lower psychological flexibility was associated with higher levels of anxiety and depression. These correlations were moderate for PPFQ-17 and strong for PPFQ-10. Concerning the three PPFQ-10 subscales; there was a significant strong negative correlation between EA and both anxiety and depression, a significant moderate negative correlation between PW and anxiety, a significant weak negative correlation between PW and depression, and furthermore significant moderate negative correlations between the VBA subscale and both anxiety and depression.

#### 3.3.2. Regression Analyses

Hierarchical regression analyses illustrated that PPFQ-10 contributed significantly to the prediction of both anxiety (*p* < 0.000) and depression (*p* < 0.000). The PPFQ-10 further accounted for 29% of the variation in HADS-a, and 35.6% of the variation in HADS-d when age, gender, and education were controlled for. Concerning the individual contributions of the PPFQ-10 subscales, VBA obtained significant beta coefficients for both anxiety and depression, with a larger unstandardized beta coefficient value for depression; EA obtained significant beta coefficients for both anxiety and depression with equally large unstandardized beta coefficient values; and PW did not obtain any significant beta coefficients for either criteria variable. The results from the hierarchical regression analyses are presented in Table 4.

## 4. Discussion

The present study assessed the adequacy of the Swedish translation of the PPFQ and evaluated the factor structure of the PPFQ-17 in a Swedish sample of 263 parents of children with chronic pain. Exploratory factor analysis with PCA supported a three-factor solution. Seven items were removed in an iterative process due to poorer extraction, significant cross loadings, or negative contributions to the overall reliability of the scale, resulting in a final version with 10 items. Notably, the three factors in the final version matched three of the four labels proposed by Wallace et al. perfectly and were retained. One of the factors, PA, was eliminated in the process, in line with author discussions in the first factor study [10]. The suggested final version of the questionnaire has three theoretically discernible subscales: VBA, PW, and (EA) [10], demonstrating fair to excellent internal consistencies. In addition, the PPFQ-10 correlated very strongly with the PPFQ-17 and was able to explain a significant amount of variance in parental anxiety and depression. Some differences regarding the subscales’ individual contributions were seen, where VBA and EA obtained significant beta coefficients, while PW did not. This result is in line with the study by Wallace et al. [10], which described the PW subscale as “the most divergent from the total”, in the sense that it had unique correlations with other main variables included in that study which the other subscales did not have. Consequently, the results from the current study are promising in their similarity to the results by Wallace et al. [10], and confirm the construct validity of the instrument when used in a different country and different language. The fact that the construct of parental psychological flexibility displays such consistency across samples and countries, points to the fact that parents’ distressing experiences in relation to their child’s pain may be of direct clinical importance. However, future studies should evaluate the psychometric properties and utility of the PPFQ-10 in different subgroups, cultures, and languages.

The levels of anxiety and depression were elevated in the current sample compared to Swedish and German normative samples [39,40]. These results are consistent with previous research on parents of children with chronic pain [41,15], and serve as yet another indication of pediatric chronic pain not only being a matter of the child, but also a family concern [42], and further point to the need for targeted parental interventions in the specific context of pediatric chronic pain. For example, earlier studies on parental distress in pediatric chronic pain have reported parents feeling a lack of life control, being caught in a pattern of short-term avoidance behaviors, and unable to change a style of parenting that they know is not appropriate in relation to their child’s age [43]. Although the correlation and regression analyses between parental distress and parental psychological flexibility in the current study had the primary objective of concurrent criteria validation, the results nevertheless provide further insight on the interrelation between these variables in parents of children with chronic pain. One possible explanation for the interrelationship seen in this study could be that lower psychological flexibility in a parent of a child with chronic pain potentially increases the risk for less adaptive and values incongruent parental behaviors, which may eventually lead to parental rumination and depression [17,43].

There are, however, a number of limitations that need to be taken into account when interpreting the findings of the current study. With a cross-sectional design, the direction of causality between parental depression and psychological flexibility remains inconclusive. A second potential restriction is that all data are based on self-report and other sources of verification of psychiatric diagnoses of parents are lacking. Further research could, for example, make use of health care records to cross-check results for parental illness. Moreover, the choice of HADS as the sole variable for concurrent criterion validation is not comprehensive, and future validation analyses of the PPFQ-10 could benefit from using a broader set of measures, including parental acceptance, parental reactivity to the child’s pain, observational data on relevant parent and child interactions, and possibly also parental cognitive flexibility and executive function. Measures of parental encouragement of the child’s illness behavior could also be helpful in establishing discriminant validity. Furthermore, some differences between the subscales’ individual contributions were seen, and the relative merits of the subscales could benefit from further examination with other samples, in outcome studies as well as in mediation analyses. Also, longitudinal or experimental designs are required to evaluate the predictive utility of the instrument for child treatment outcomes, and also to establish if treatment interventions specifically targeted at increasing parental psychological flexibility may ultimately benefit both parent and child outcomes.

In conclusion, the factor structure and reliability of this revised and shortened version of the original PPFQ are supported within our results. Results also illustrate the importance of the PPFQ-10 in explaining variance in parental anxiety and depression, supporting criterion validity. Based on the existing data, the PPFQ-10 could serve as a useful tool to assess psychological inflexibility in parents of children with chronic pain, simultaneously reducing the burden of research and assessment.

## Figures and Tables

**Table 1:** Factor loadings and communalities, based on a principle components analysis (PCA) with oblimin rotation, for 10 items from the 17-item version of The Parent Psychological Flexibility Questionnaire (PPFQ-17; *n* = 263).

Item No. ^a^	Item ^a^	Factor Loadings ^b^			
		EA	PW	VBA	Communality (Extraction)
7	Despite my child’s pain, we are able to pursue activities that are important to our family.		0.157	0.820	0.721
8	When my child has pain episodes, I am able to remain aware of our goals and other things that are important to us as a family.	0.271	−0.113	0.715	0.696
11	It is possible to live a normal life while my child suffers with pain.			0.814	0.652
9*r*	I avoid situations where my child will have pain.		0.756	0.124	0.607
13*r*	Pain control must come first whenever my child does activities.		0.780	−0.138	0.698
24*r*	My child must avoid activities that lead to pain.		0.826		0.683
22*r*	I suffer terribly from my child’s pain and need to make this suffering stop.	0.830			0.744
26*r*	My child’s pain makes it impossible to focus on anything else.	0.683		0.243	0.717
28*r*	I am overwhelmed by worry over my child’s pain.	0.845			0.753
31*r*	I struggle with my own thoughts and feelings about my child’s pain.	0.853			0.681

^a^ From the original PPFQ by McCracken et al. [11]; *r* = reversed scored item. Values are reported from the pattern matrix, sorted by subscale; total variance explained = 69.5%; Factor loadings < 0.1 are suppressed; ^b^ Extraction method: PCA, with oblimin as the rotation method (kaiser normalized; rotation converged in five iterations); EA, emotional acceptance; PW, pain willingness; VBA, values-based action.

**Table 2:** Descriptive statistics for the 10-item PPFQ (PPFQ-10) subscales and full scale (*n* = 263).

Scale	No. of Items	Mean (*SD*)	Skewness	Kurtosis	Alpha
VBA	3	10.41 (*4.06*)	−0.208	−0.363	0.76
PW	3	7.23 (*4.32*)	0.269	−0.698	0.73
EA	4	12.48 (*5.97*)	0.166	−0.741	0.87
Full scale	10	30.12 (*11.45*)	−0.059	−0.306	0.86

**Table 3:** Descriptive statistics and correlations between controls and variables of sample (*n* = 263).

Variables	Mean (%)	*SD*	Age	Gender	Education	PPFQ-17 Total	PPFQ-10 Total	PPFQ-10 VBA	PPFQ-10 PW	PPFQ-10 EA	HADS-a
1. Age	44.4	6.5									
2. Gender (F, M)	(81, 19)	N/A	−0.22 **								
3. Education (Hi, Lo)	(53, 47)	N/A	0.24 **	0.05							
4. PPFQ-17 total	47.0	18.0	0.09	0.01	0.19 **						
5. PPFQ-10 total	30.1	11.5	0.08	0.04	0.19 **	0.96 **					
6. PPFQ-10 VBA	10.4	4.0	0.00	0.02	0.11	0.070 **	0.76 **				
7. PPFQ-10 PW	7.2	4.3	0.11	0.10	0.21 **	0.71 **	0.73 **	0.34 **			
8. PPFQ-10 EA	12.5	6.0	0.08	−0.01	0.14 *	0.86 **	0.88 **	0.53 **	0.44 **		
9. HADS-a	6.1	4.5	−0.06	0.11	−0.06	−0.48 **	−0.52 **	0.39 **	−0.30 **	−0.52 **	
10. HADS-d	4.4	4.0	0.03	0.01	−0.04	−0.49 **	−0.53 **	−0.49 **	−0.23 **	−0.53 **	0.72 **

Variables above the line, controls; variables below the line, predictors in the regression analyses; HADS-a, The Hospital Anxiety and Depression Scale—anxiety; HADS-d, The Hospital Anxiety and Depression Scale—depression; * Correlation is significant at the 0.05 level (two-tailed); ** Correlation is significant at the 0.01 level (two-tailed).

**Table 4:** Hierarchical regression analysis to evaluate the criteria validity of the PPFQ-10.

Criteria	Step	Predictor Variables	*R*^2^	*R*^2^ Change	*F* Change (*df*)	Sig. *F* Change	Unstandardized ^a^		Standardized Coefficients Beta ^a^		
							B	SE	β	t	Sig.
**HADS-a**	1	Control variables	0.013	0.013	1.104 (3252)	0.348					
		Age					−0.001	0.038	−0.001	−0.021	0.983
		Gender					1.203	0.626	0.106	1.921	0.056
		Education					0.283	0.493	0.032	0.575	0.566
	2	PPFQ-10	0.303	0.290	34.512 (3249)	0.000					
		VBA					−0.182	0.070	−0.168	−2.621	0.009
		PW					−0.094	0.062	−0.092	−1.505	0.134
		EA					−0.287	0.051	−0.385	−5.685	0.000
**HADS-d**	1	Control variables	0.002	0.002	0.195 (3252)	0.900					
		Age					0.044	0.034	0.072	1.30	0.20
		Gender					0.489	0.550	0.048	0.889	0.375
		Education					0.478	0.435	0.060	1.098	0.273
	2	PPFQ-10	0.358	0.356	46.003 (3249)	0.000					
		VBA					−0.310	0.060	−0.318	−5.184	0.000
		PW					0.007	0.053	0.008	0.131	0.896
		EA					−0.246	0.043	−0.369	−5.671	0.000

Results show the amount of variance explained by PPFQ-10 in anxiety and depression (criteria variables); ^a^ Results are displayed from the final model; Sig., significance.
